# Recent advances in the treatment of *Pseudomonas aeruginosa *infections in cystic fibrosis

**DOI:** 10.1186/1741-7015-9-32

**Published:** 2011-04-04

**Authors:** Niels Høiby

**Affiliations:** 1 Department of Clinical Microbiology 9301, Rigshospitalet & ISIM, University of Copenhagen, Juliane Maries vej 22, DK-2100 Copenhagen, Denmark

## Abstract

Chronic *Pseudomonas aeruginosa *lung infection in cystic fibrosis (CF) patients is caused by biofilm-growing mucoid strains. Biofilms can be prevented by early aggressive antibiotic prophylaxis or therapy, and they can be treated by chronic suppressive therapy. New results from one small trial suggest that addition of oral ciprofloxacin to inhaled tobramycin may reduce lung inflammation. Clinical trials with new formulations of old antibiotics for inhalation therapy (aztreonam lysine) against chronic *P. aeruginosa *infection improved patient-reported outcome, lung function, time to acute exacerbations and sputum density of *P. aeruginosa*. Other drugs such as quinolones are currently under investigation for inhalation therapy. A trial of the use of anti-*Pseudomonas *antibiotics for long-term prophylaxis showed no effect in patients who were not already infected. Use of azithromycin to treat CF patients without *P. aeruginosa *infection did not improve lung function. Here I review the recent advances in the treatment of *P. aeruginosa *lung infections with a focus on inhalation treatments targeted at prophylaxis and chronic suppressive therapy.

## Review

Cystic fibrosis (CF) is a congenital, recessively inherited disorder which affects one of 2,000 newborns in Caucasian populations. In Europe, approximately 35,000 children and young adults have CF. The prevalence in the USA and Canada is approximately 30,000 and 3,000, respectively. CF is also found in the Australia, New Zealand, the Middle East, Iran, Pakistan, India and Latin America. If not treated, most CF patients die at a young age. If intensively treated, the mean expected lifetime of CF patients is >35 years, and in some centres it is >50 years. Any progress in treatment is therefore important for CF patients. The genetic background is >1,500 mutations in the cystic fibrosis transmembrane conductance regulator gene (*CFTR*) on chromosome 7 which lead to malfunction of the chloride channel in CF patients. The disease affects the airways, the pancreas, the small intestine, the liver, the reproductive tract and the sweat glands. The clinical symptoms are viscid mucus, respiratory infections, intestinal malabsorption of fat, diabetes mellitus, meconium ileus, impaired liver function, male infertility and salt loss. The increased concentration of sodium chloride in the sweat is used for the diagnosis of CF.

The malfunction of the chloride channel in CF patients leads to decreased volume of the paraciliary fluid in the lower respiratory tract, and that in turn leads to impaired mucociliary clearance of inhaled microbes [1]. This impairment of the noninflammatory defence mechanism of the respiratory tract leads to early recruitment of the inflammatory defence mechanisms such as polymorphonuclear leukocytes (PMN) and antibodies [2-4]. Therefore, from early childhood, CF patients have recurrent and chronic respiratory tract infections characterised by PMN inflammation. In spite of the inflammatory response and intensive antibiotic therapy, however, infections caused by *Pseudomonas aeruginosa*, the *Burkholderia cepacia *comple*x *(mostly *B. multivorans *and *B. cenocepacia*) and *Achromobacter xylosoxidans *persist and lead to respiratory failure and lung transplantation or death [5]. Several other species, including *Staphylococcus aureus*, *Haemophilus influenzae*, *Stenotrophomonas maltophilia *and *Mycobacteria *other than tuberculosis and *Aspergillus fumigatus*, also contribute to morbidity and mortality in CF patients [5]. Chronic *P. aeruginosa *lung infection is the cause of much of the morbidity and most of the mortality in CF patients. About 80% of adults with CF have chronic *P. aeruginosa *infection. Previously, 50% of CF patients would die within 5 years after the onset of the chronic *P. aeruginosa *infection, but intensive early eradication therapy has completely changed the prognosis, and most patients therefore do not contract the chronic infection during childhood anymore [6].

Adaptive mechanisms of *P. aeruginosa *exist, which explains why this pathogen is able to survive and persist for several decades in the respiratory tract of CF patients in spite of the defence mechanisms of the host and intensive antibiotic therapy. *P. aeruginosa *is able to survive by switching to the biofilm mode of growth, which provides tolerance to the inflammatory defence mechanism, to the aerobic respiratory zone and to the conductive zone of the lungs which contain anaerobic sputum, and tolerance to antibiotic therapy [7-10]. During the adaptation, mucoid (biofilm mode of growth) and nonmucoid phenotypes are split off due to mutations (Table 1). The biofilm strategy is also used by *Burkholderia*, *Achromobacter *and *Stenotrophomonas *species [11].

**Table 1:** Important properties of mucoid and nonmucoid phenotypes of *Pseudomonas aeruginos a *in the respiratory tract of cystic fibrosis patients^a^

Property	Mucoid phenotype	Nonmucoid phenotype
Location in the lungs	Respiratory zone and conductive zone in sputum	Conductive zone in sputum
Biofilm formation *in vitro*	Yes	Yes
Biofilm formation *in vivo*	Yes	No
Multiply antibiotic resistance due to conventional mechanisms	Seldom	Frequent
Resistance (tolerance) due to biofilm properties	Yes	No
Responsible for lung tissue damage	Yes	No
Induces pronounced antibody response	Yes	No

^a^Table 1 is based on data in references [9] and [10].

### Current strategies for management of bacterial infections in cystic fibrosis

**Figure 1:**
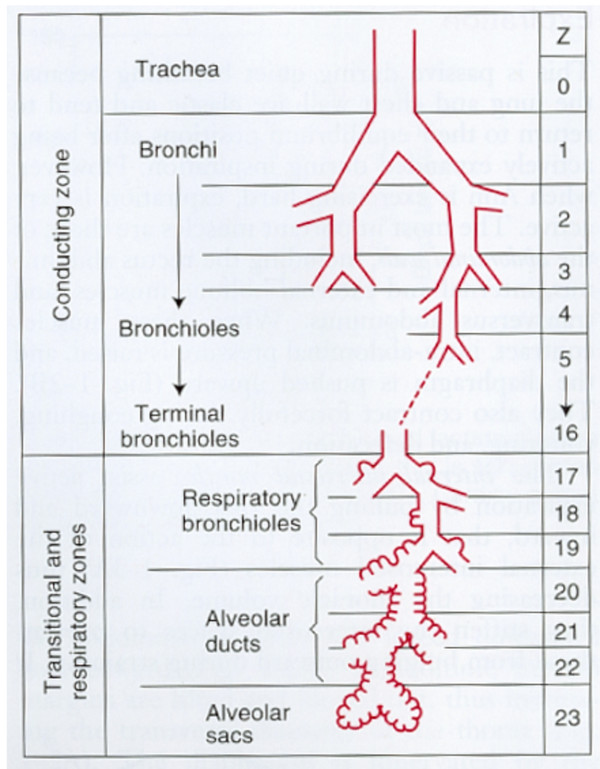
**The conductive and respiratory zones of the lungs **[12]. Inhalation antibiotic therapy mainly targets the conductive zone where sputum is located, whereas systemic antibiotic therapy mainly targets the respiratory zones with no sputum [9,10].

The lungs consist of the smaller conductive zone and the larger respiratory zone (Figure 1 and Table 1). The respiratory zone includes respiratory bronchioles, alveolar ducts and alveolar sacs [10,12,13]. This part of the lungs has no cilia, no goblet cells and no submucosal glands, and the defence system includes alveolar macrophages and defensins. All the venous blood of the body passes through the capillaries of the alveolus, which consist of a nearly continuous sheet of blood, and only a very thin barrier is present between the air and the blood. The smaller conducting zone includes the trachea, the bronchi and the terminal bronchioles. This part of the lungs has cilia, goblet cells and submucosal glands and has an ordinary arterial blood supply from the aorta. The mucus is produced in the respiratory zone, and the major defence system is composed of the mucociliary escalator [12] and PMNs recruited from the respiratory zone [14]. Nebulised tobramycin and colistin and other antibiotics are widely used to treat *P. aeruginosa *lung infection in CF patients. Very high concentrations of these drugs are obtained in the conductive zone (sputum), whereas very little actually reaches the respiratory zone, since the measurable concentration in serum, which reflects the amount in the respiratory zone, is very low [15-19]. On the contrary, when antibiotics are administered intravenously or orally, very low concentrations are found in sputum, but high concentrations are found in the respiratory tissue because the whole dose of, for example, an intravenous (i.v.) bolus of antibiotics is transported directly by the blood to the alveolar capillaries before being distributed to the rest of the body [20]. Since both the respiratory and the conductive zones of the lungs are infected with *P. aeruginosa *[9,21,22], there is both *in vitro *and *in vivo *pharmacokinetic and pharmacodynamic evidence for using combined systemic and nebulised antibiotics in CF patients [23-25]. Most of the research in antibiotic management of bacterial infections in CF is carried out either in intermittently colonised patients to prevent chronic infection or in chronically infected patients to prevent further tissue damage in the respiratory zone. These current therapeutic strategies are described in consensus reports [26,27]. However, the burden of treatment for CF patients is large, and adherence to therapy may therefore be difficult, so there is a great need for novel therapeutic strategies.

## Recent trials and results

### Inhaled medications and inhalation devices

The size of the nebulised droplets and particles carrying the antibiotic are important with regard to where they are deposited in the lungs. The optimal particle size for reaching the small airways is 1 to 5 μm; smaller droplets and particles will not be deposited and larger ones will only reach the upper airways [28].

The particle size (1 to 5 μm), the lung dose delivered (a range between 3% and 8% up to >50%) and the administration time (10 to 20 minutes to <2 or 3 minutes) have all improved during recent years as treatment of lung infections with antibiotics administered by inhalation therapy has become standard care in CF centres. This important development means that some antibiotics, such as colistin, tobramycin and aztreonam can now be inhaled within 2 to 3 minutes one to three times daily. The evolution of therapy from jet nebulisers to dry powder inhalers and pressured metered dose inhalers, as well as the relevant publications, are summarized in two excellent recent surveys [28,29]. The most important advance of dry powder inhalation is that the time required for delivering each dose is less than one-third the time required for nebulisation, and this fact is expected to improve patients' adherence to therapy [30,31], since there is no immediate relief after antibiotic inhalation, it is time-consuming and it makes CF visible [32].

### Inhaled versus systemic antibiotics for early eradication therapy

Inhaled as well as systemic antibiotic administration, either alone or combined, is widely used in CF patients. Inhaled tobramycin has been shown to transiently clear *P. aeruginosa *from the lower airways in CF patients but does not markedly reduce lung inflammation, which is a key factor in disease progression [33]. Clinically stable children with CF (from 0.5 to 16 years of age) with recent *P. aeruginosa *infection were therefore randomised to receive four weeks of inhaled tobramycin or two weeks of systemic i.v. ceftazidime and tobramycin. If i.v. treatment was not possible, oral ciprofloxacin and nebulised tobramycin for two weeks was used. Bronchoalveolar lavage fluid (BALF) was obtained just before and four to six weeks after treatment. It was considered impossible to blind the study, but measurement of inflammatory parameters was done blindly. The primary outcome was the change in the percentage of PMNs in BALF from the worst lobe, and secondary parameters were changed in total cells, PMNs, cytokines and bacterial quantity. Only 21 of 41 patients agreed to be enrolled in the study, and only 15 patients completed the study (six in the inhalation group and nine in the systemic group). The study was therefore underpowered, but this may not be the only explanation why most of the results were nonsignificant. However, median changes in total cells per millilitre and PMNs per millilitre BALF were substantially greater in the systemic group than in the inhalation group (*P *< 0.01 and *P *< 0.02, respectively). The greatest decrease was found in the patients treated with both ciprofloxacin and tobramycin, whereas the inhalation group showed little change. What is still needed is a large multicentre study in which oral ciprofloxacin combined with nebulised tobramycin or colistin is compared with nebulised tobramycin or colistin using the same primary and secondary parameters [33].

### *Nebulised aztreonam lysine for *P. aeruginosa *infection*

The major effects of inhaled antibiotics are improvement of lung function by suppression of chronic lung infection or eradication of intermittent colonisation to prevent chronic lung infection. The major risks are local side effects, such as coughing, inflammation, allergy and development of resistance to the antibiotic. The only approved antibiotics for inhalation therapy in CF have for a long time been colistin and tobramycin, but the development of antibiotic resistance, for example, has promoted off-label use of other antibiotics in CF centres. New approved antibiotics for inhalation therapy are therefore needed.

Three large, multinational, multicentre phase III studies (AIR-CF1 through AIR-CF3) of the use of nebulised aztreonam lysine (a monobactam antibiotic with Gram-negative spectrum) have recently been published [34-36] (Table 2). They showed that nebulised 75 mg of aztreonam lysine is a safe and efficient treatment which can be used repeatedly to suppress the chronic *P. aeruginosa *lung infection in CF patients. The end points of the study were patient-reported outcomes, improvement of forced expiratory volume in one second (FEV_1_) and decreases of *P. aeruginosa *density in sputum, the time needed for inhaled or i.v. antipseudomonal antibiotics and the time to acute exacerbations. Comparable results were obtained with the twice daily and three times daily dosing regimens [34,35]. Adverse events were the same in the placebo and aztreonam arms of the study and were consistent with CF lung disease. A transient fourfold increase in minimum inhibitory concentrations was found in the aztreonam-treated patients.

**Table 2:** Some recent publications on inhalation therapy in cystic fibrosis^a^

Topic	Reference
General aspects of inhalation therapy	[28]
Dry powder inhalers	
Tobramycin	[30]
Colistin	[31]
Aztreonam lysine for inhalation	
AIR-CF1 aztreonam three times daily versus placebo	[34]
AIR-CF2 aztreonam twice daily versus three times daily versus placebo	[35]
AIR-CF3 follow-up of AIR-CF1 and AIR-CF2 with repeated aztreonam courses	[36]
Fosfomycin in combination with tobramycin	[45]

The patients enrolled in AIR-CF1 and AIR-CF2 were then followed for 18 months in the open-labelled AIR-CF3 study, in which up to nine repeated 28-day courses of twice daily and three times daily administration of aztreonam were given, alternating with 28 days off treatment [36]. Generally, the results from the AIR-CF1 and AIR-CF2 studies were replicated in the AIR-CF3 study, but the three times daily dosing regimen produced significantly better results than twice daily dosing in terms of lung function and respiratory symptoms. Adherence to treatment was 92% in the twice daily group and 88% in the three times daily group [36].

### *Cycled antibiotic prophylaxis to prevent *P. aeruginosa *colonisation*

Prevention of chronic lung infection caused by *P. aeruginosa *and other microorganisms is cost-effective and prolongs the lives of CF patients [37,38]. Early, aggressive eradication of intermittent *P. aeruginosa *colonisation is a treatment that has to be repeated to keep most of the patients free of chronic *P. aeruginosa *infection [6]. A triple-blind, placebo-controlled, randomised, three-year study was therefore carried out to investigate whether three-week treatments administered every three months with oral ciprofloxacin and inhaled colistin, compared with placebo would prevent initial *P. aeruginosa *infection [39]. The primary outcome was *P. aeruginosa *infection, and therefore respiratory cultures were obtained every three months. The percentage of cultures containing nonfermenting, Gram-negative bacteria other than *P. aeruginosa *was significantly higher in the treatment group, and treatment administered once every three months did not reduce the risk of initial and chronic *P. aeruginosa *infection.

### *Effect of azithromycin on pulmonary function in CF patients without *P. aeruginosa *infection*

Azithromycin is used routinely in the treatment of CF patients with chronic *P. aeruginosa *infection, since a number of trials in adults and children with CF showed improvement in lung function [40]. The mechanism by which azithromycin works is thought to be through inhibition of bacterial communication (quorum sensing) and reduction of inflammation, since azithromycin does not inhibit the growth of *P. aeruginosa *at the concentrations obtainable *in vivo*. This was the rationale behind a large multicentre, randomised, double-blind, placebo-controlled trial in which azithromycin (250 mg or 500 mg depending on body weight) was given every other day to children or young adults with CF who had negative *P. aeruginosa *cultures for at least one year. The primary outcome was change in FEV_1 _[41]. Twenty-four weeks of treatment with azithromycin did not result in improved pulmonary function, but the treated group had a reduction in exacerbations and cough compared with the placebo group. On the other hand, significantly more macrolide-resistant *S. aureus *and *H. influenzae *were isolated in the treated group.

#### Ongoing studies

At present, there are two multicentre, randomised, double-blind, placebo-controlled studies with a focus on the prevention of chronic *P. aeruginosa *infection by early, aggressive eradication. The American multicentre EPIC study [42] comprising 300 patients ages one to 12 years has four arms in which 28 days of four different treatment inhalation strategies are compared. Two culture-based therapies are being administered in this study (1) inhaled tobramycin and oral placebo and (2) inhaled tobramycin and 14 days of oral ciprofloxacin, and the two non-culture-based cycled therapies (3) inhaled tobramycin and oral placebo and (4) inhaled tobramycin and 14 days oral ciprofloxacin. Therapies (1) and (2) are repeated when quarterly respiratory cultures are found positive for *P. aeruginosa*, and (3) and (4) are followed by 56 days off therapy for six quarterly cycles. The preliminary results [43] showed no significant differences between the four strategies with respect to either *P. aeruginosa*-positive cultures or exacerbations, and there were no concerns regarding drug toxicity or development of resistance.

The Scandinavian multicentre study (200 patients) compared the standard three-week nebulised colistin and oral ciprofloxacin treatment and additional oral azithromycin or placebo for the prevention of chronic *P. aeruginosa *infection [44]. This is a culture-based study, and in addition the patients are screened for *P. aeruginosa *antibodies to be sure that the patients do not have chronic infection at the time of enrollment. The rationale of the study is the demonstrated quorum-sensing inhibitory effect of azithromycin, which could disarm *P. aeruginosa *and thereby facilitate its early eradication [7]. The study is ongoing, and no results have been reported.

#### The biofilm mode of growth: the major hurdle for the future

The major hurdle is the biofilm mode of growth of *P. aeruginosa *and also *Burkholderia cepacia complex*, *Achromobacter xylosoxidans *and *Stenotrophomonas maltophilia*, which have become established CF pathogens resistant to eradication with antibiotics [5,11]. Since a major side effect of antibiotic maintenance therapy is development of resistance to the antibiotics by means of conventional resistance mechanisms, new antibiotic formulations for inhalation therapy are needed. An interesting approach is the combination of fosfomycin and tobramycin, which has been investigated in a phase II study with promising results in CF patients with *P. aeruginosa *infection [45]. At present, we do not know much about the pharmacokinetics and pharmacodynamics of antibiotics and biofilms [46-48], and this topic should be studied in detail in the future. Likewise, the synergy obtained by combination therapy should be further studied, since recent results indicate that the combination of tobramycin and colistin is superior to single-drug therapy *in vitro*, in animal experiments and in a pilot nebulisation study in CF patients [49]. Combination therapy utilising ciprofloxacin and colistin has also shown synergy *in vitro*, since the surfaces of biofilm-growing bacteria are killed by ciprofloxacin, whereas those in the deeper layer are killed by colistin [23,50]. Use of nebulised deoxyribonuclease (DNase) seems to reduce the incidence of new infections in CF patients [51], and the optimal combination with antibiotics needs to be studied. There are several experimental possibilities which should be tested in the future, including inhibition of quorum sensing by, for example, garlic [52], or breaking of the biofilm matrix by, for example, alginate lyase, F-actin or DNase [53].

## Conclusions

New formulations of old antibiotics (aztreonam) used to treat chronic *P. aeruginosa *infection have been shown to be effective and valuable additions to the current strategy of maintenance treatment of CF patients [52,53]. Future studies will hopefully explore the question whether combination therapy with other inhaled antibiotics is more efficient than current treatment strategies. Several other drugs, such as quinolones and amikacin in liposomal formulation, are currently under investigation for inhalation therapy, and they are needed because of resistance to the traditional antibiotics. Furthermore, the above-mentioned *in vitro *studies indicate that alternative strategies may be used clinically in the future to combat biofilm infections in CF patients. The scientific activity in CF care is very high because of the severity of the disease and because of centralized treatment and widespread international cooperation between CF centres [54]. CF patients may in this respect be regarded as pioneers for other patients with severe chronic lung infections, for whom the described therapeutic principles may also be beneficial.

## Abbreviations

BALF: bronchoalveolar lavage fluid; CF: cystic fibrosis; CFTR: cystic fibrosis transmembrane conductance regulator; FEV_1_: forced expiratory volume in 1 second; DNase: deoxyribonuclease; i.v., intravenous; PMN: polymorphonuclear leukocytes.

## Competing interests

The author declares that he has no competing interests.

## Pre-publication history

The pre-publication history for this paper can be accessed here:

http://www.biomedcentral.com/1741-7015/9/32/prepub
